# Platform-Based Internationalization of Smaller Firms: The Role of Government Policy

**DOI:** 10.1007/s11575-022-00492-z

**Published:** 2022-11-26

**Authors:** Nitish Singh, Surender Munjal, Sumit Kundu, K. Rangarajan

**Affiliations:** 1 David Orthwein Endowed Professor of International Business, Richard A. Chaifetz School of Business, Saint Louis University St. Louis, St. Louis, MO 63108 USA; 2 Professor of International Business and Management, Leeds University Business School, University of Leeds, Leeds, LS2 9JT UK; 3 James K. Batten Eminent Scholar Chair in International Business, Department of International Business, College of Business Administration, Florida International University, Miami, FL 33199 USA; 4 Founder Head - Centre for MSME Studies, and Head – IIFT Kolkata Campus Indian Institute of Foreign Trade, Kolkata, West Bengal 700107 India

**Keywords:** E-Commerce, Export, India, Small and Micro Enterprises, Policy, Platforms

## Abstract

Governments are recognizing anticompetitive concerns and market distortions associated with the rise of e-commerce platforms. Thus, policies are being crafted to level the playing field between large platform operators and small platform sellers. In addition, policies mitigating barriers to internationalization associated with using e-commerce platforms are also being developed. This study aims to explore these policy actions impacting the platform-based internationalization of small and micro enterprises (SMEs). The case study method is adopted to provide rich insights into the Indian Government policy actions impacting the e-commerce platform-based internationalization of Indian SMEs. More specifically, this investigation analyzes the extent to which the Indian Government policy actions have mitigated the home market and industry-level barriers to export for SMEs leveraging e-commerce platforms for their internationalization efforts. Study findings provide rich policy implications and fodder for future empirical testing of key qualitative insights uncovered in this study.

**Supplementary Information:**

The online version contains supplementary material available at 10.1007/s11575-022-00492-z.

## Introduction

E-commerce platforms^1^ are increasingly facilitating small businesses' internationalization (Manyika et al., 2016; OECD, 2019). COVID-19 concerns are also leading businesses to embrace e-commerce platforms for the expeditious transition of offline undertakings to online activities (Chen, 2020). According to an industry report, online marketplaces generated $1.7 trillion in 2019, accounting for 50% of e-commerce sales worldwide (Merton, 2021). In response to this rapid rise of e-commerce platforms or marketplace operators, governments are crafting policies to ensure that these platforms provide safeguards against unfair trading practices and do not monopolize markets due to their network-effects (Nambisan et al., 2019). One of the main concerns of various governments is protecting marketplace sellers from anticompetitive practices on online platforms. For example, many smaller sellers on large e-commerce platforms face challenges such as listing visibility, customer review manipulations, competition from platforms own products/brands, platforms one-sided access to customers’ and sellers’ data, account suspensions, and *black-hat* tactics by other sellers (European Commission, 2020; McKnight, 2020). Indeed, these challenges are more pronounced for smaller firms, particularly in emerging economies, due to their weak resource endowments and limited negotiation power with the E-commerce platforms.

While policies are evolving to protect small sellers and regulating online marketplaces in economically advanced countries (e.g., Digital Single Market Initiative of the European Commission, 2020), it is salient for emerging economies too with a large base of smaller enterprises to update their institutional frameworks for managing platform-based economy's competitive dynamics. India has made an important step in this direction by proposing an E-commerce Policy (Government of India, 2019a) to strengthen its institutional environment. India’s policy casts a wide net with measures covering competitive parity and mitigating large e-commerce platforms' coercive power, and concatenating steps focused on incentivizing, educating, and empowering small sellers to efficiently leverage e-commerce for their internationalization efforts.

With this backdrop, the current study aims to explore how government policies impact platforms-based internationalization of ‘small and micro enterprises’ or SMEs. It focuses on the Indian Policy context as India is one of the few emerging economies that has undertaken significant policy developments, addressing SMEs' internationalization via e-commerce platforms. With its proclaimed aim, this study contributes to this special issue on ‘E-Commerce Policy and International Business’ (Cummings et al., 2020) and the evolving body of literature that examines the role of institutions in promoting the firm’s internationalization via e-commerce platforms.

There is a vast literature supporting institutions' role in firms' internationalization (Leonidou et al., 2011; Peng, 2003; Peng et al., 2009). Past studies in international business (IB) have also investigated e-business-related internationalization, the role of e-commerce in internationalization, and extended IB theories to explain the e-business internationalization phenomenon (e.g., Brouthers et al., 2016; Chen et al., 2019; Jean & Kim, 2020; Singh & Kundu, 2002; Zeng et al., 2019). However, there is a dearth of studies exploring internationalization at the intersection of institutions and e-business/e-commerce platforms.

Recent studies have started to investigate the role and importance of e-commerce platforms in the IB literature (e.g., Brouthers et al., 2016; Chen et al., 2019; Jean & Kim, 2020; Jean et al., 2020; Nambisan et al., 2019; Ojala et al., 2018; Stallkamp & Schotter, 2021; Zeng et al., 2019). However, most of these studies have focused on the internationalization of e-commerce platform firms and the associated value creation, strategic, and technical issues (Ojala et al., 2018; Stallkamp & Schotter., 2021; Zeng et al., 2019). This is particularly surprising because platform-based internationalization is increasingly gaining momentum. Simultaneously, policies are catching up to address associated challenges, but scholarly research has not kept pace with this growing phenomenon (Cummings et al., 2020; Jean & Kim, 2020).

To systematically analyze the role of policy and provide a relevant context for our analysis, we focus on Indian Governmental policy measures to enhance local SMEs platform-based internationalization efforts. We classify these policy measures based on the internationalization barriers they aim to mitigate. Consequently, we investigate home-market and industry-level barriers that are targeted in the Indian Government’s policies in order to enhance Indian SMEs platform-based internationalization. Given the paucity of research in this area, we utilize a more nuanced epistemological approach based on case studies to help us identify context-rich insights to describe the impact of Indian Government policies on the platform-based internationalization of Indian SMEs.

Research Question-1: What is the impact of the Indian Government policies on mitigating home country-level market barriers to SME internationalization using e-commerce platforms?

Research Question-2: What is the impact of the Indian Government policies on mitigating industry-level market barriers to SME internationalization using e-commerce platforms?

## E-Commerce Platforms and the Institution-Based View

In the IB literature, digital platforms have been generally conceptualized as being multi-sided in nature and thus mediating between different user groups (Brouthers et al., 2016; Zeng et al., 2019). IB researchers have also emphasized platforms' unique capabilities of aggregation, network externalities, shared platform resources, complementation, modularity, governance, and value creation (Brouthers et al., 2016; Jean & Kim, 2020; Nambisan et al., 2019; Zeng et al., 2019). In this study, we conceptualize the role of the e-commerce platform as the facilitator of SME internationalization. Thus, we emphasize the platforms' multi-sided nature of connecting buyers and sellers from different countries. Hence, we define e-commerce platforms as "*online marketplace operators bringing together many buyers and sellers by leveraging information, technology, governance mechanisms, and complementors to enable exchange and value creation in a global arena*."

Studying SMEs’ internationalization via e-commerce platforms without considering the institutional forces impacting these online marketplaces may not fully explain the underlying dynamics of internationalization in this context. Ultimately, SMEs owners' decisions to leverage e-commerce platforms for internationalization is not only the result of the entrepreneurs’ decision-making but is also influenced by various policies and actions by governments. Admittedly, according to Clegg (2019), all modalities and outcomes explored in IB result from private decision-making and policy actions. Indeed, researchers have called for the exploration of country-specific institutions' role in shaping the dynamics of global competition and internationalization resulting from the proliferation of electronic markets (Grewal et al., 2001; Nambisan et al., 2019). Furthermore, the network effects associated with large platform companies such as Amazon and eBay are compounding the winner-take-all economy, resulting in regulatory responses to curb the rise of such monopolistic advantages (Nambisan et al., 2019). Therefore, it is no surprise to see the rise of policy initiatives worldwide attuned to regulating e-commerce platforms and associated ecosystems of buyers, sellers, and complementors. Policies are also being fashioned to enable SMEs to participate in the global digital economy. For example, India’s Digital MSME (Micro, Small, and Medium Enterprises) guidelines empower and educate businesses to use online technology to access national and international markets. Therefore, this study considers the role of government policy and actions to be an essential catalyst for SMEs’ internationalization via e-commerce platforms. Hence, we lean on the institution-based view for understanding government actions and platform-actors' interaction and the resulting strategic outcomes of such interaction.

Past studies have emphasized the role of institutions in constraining, facilitating, or supporting firms' growth and internationalization (Kahiya, 2018; Scott, 2001). Dimaggio and Powell (1983) argue that institutions exert isomorphic pressures on organizations by prescribing what actions are acceptable and not, which shapes a firm’s behavior and structure. This view of the institutional theory perceives firms as passive receivers, reacting to the evolutions in the given institutional environment and surviving by legitimizing their actions. However, scholars (Cantwell et al., 2010; McGaughey et al., 2016) argue that institutions also co-evolve with the firm’s evolution. Such institutional evolution is often triggered by changes in prevailing industry dynamics, technology, and novel business models. Peng et al. (2017) emphasize that both firms and governments need to encourage institutional change in response to this age of accelerated growth, trade, and technological discontinuity. The resulting institutional transitions in the formal and informal rules governing the interactions of governments and organizations (Peng, 2003) reduce uncertainty and lead to institutional development for higher economic growth (Peng et al., 2017).

This dynamism in the institutional evolution has been articulated by Clegg (2019, p. 113), wherein he argues that "while in many ways formal institutions reflect a sense of stability and permanence [to provide durable social structures as articulated by (Scott, 2001)], part of their purpose is also to respond in a timely manner to new information and new situations." Clegg contends that governments often devise policies to deal with new realities, and these policies later pave the way for the formation of new institutions or amendments to old institutions.

In the e-commerce platform context, governments face many new realities, which require adapting or proposing new policies and actions. According to a report by the United Nations Conference on Trade and Development (UNCTAD), e-commerce platforms, unlike traditional organizations, pose complexities of digital transformation related to information and communications technology (ICT) infrastructure, logistics, e-transactions, use of third-party services, and seller and consumer data issues (UNCTAD, 2019). These complexities are further hyperbolized due to platform-based idiosyncratic issues such as network effects, value-creation based on data and data interactions, disruptive innovation, scale without mass,^2^ and possible economies of scope (Brynjolfsson et al., 2008; OECD, 2019; UNCTAD, 2019). Appropriately, these platform-centric complexities create policy challenges regarding fast-evolving issues such as cross-border data flow, data ownership and control, data privacy and protection, business competencies for leveraging platform-based technologies, and anticompetition issues (UNCTAD, 2019). Hence, governments need to gather market intelligence to adapt or propose new policies and actions for constantly evolving platform-centric issues.

The following section looks at how government policy and actions may assist SMEs in internationalizing using e-commerce platforms. To enrich this discussion, we contextualize policy actions by elaborating on Indian Governmental policies impacting local SME internationalization via e-commerce platforms.

## Indian Government Policies Impacting Platform-Based Internationalization of SMEs

E-commerce platforms with global reach, affordability, efficiency, brand recognition, exporting know-how, and third-party services enable accelerated internationalization of small businesses (Jean & Kim, 2020; Manyika et al., 2016; Nambisan et al., 2019). Nevertheless, there are several ways platform operators can exercise inordinate influence over sellers. Platform operators could use unfair practices such as leveraging customer and seller data to directly compete with their sellers, not being transparent about ranking criteria, having complex terms and conditions, retroactively changing terms and conditions, and suspending seller accounts (European Commission, 2020). However, government policies could help negotiate the balance of power between the platform operator and the SME seller and manage anticompetition concerns. Government policies can also help SMEs by mitigating and removing barriers related to export procedures and other administrative hurdles.

Furthermore, government policies can help educate and train SMEs to leverage platform-based technology and export knowledge for global growth. Thus, we specifically investigate home-market and industry-level barriers (Kahiya, 2013, 2018; Leonidou, 2004; Tesfom & Lutz, 2006) that Indian Government policies and actions may mitigate to enhance Indian SMEs’ platform-based internationalization.

### Indian Government Policies Mitigating Home Market Barriers

Home market barriers impacting a firm's internationalization include issues such as infrastructure, export documentation, exporting knowledge, export payments, banking support, taxes, logistics, export incentives, and export promotion programs (Kahiya, 2013, 2018; Leonidou, 2004; Leonidou et al., 2011; Tesfom & Lutz, 2006). These home market barriers impact internationalizing firms' operational efficiency, communication effectiveness, and the ability to transact with foreign customers (Leonidou, 2004). Thus, government policies and related export assistance programs to mitigate these barriers can help firms' internationalization efforts. Accordingly, we classify policy responses under three subcategories: a) policies enhancing export efficiency, b) policies mitigating e-commerce governance-related concerns, and c) policies mitigating export knowledge and skill barriers.

#### Policies Enhancing Export Efficiency

This subcategory includes India's National E-commerce Policy (Government of India, 2019a) inputs regarding building digital infrastructure and assuaging barriers related to export procedures, logistics, payments, banking, technology, data access, and more. For example, this policy takes forward the Digital India Initiative; the goal is to develop secure and stable digital infrastructure able to provide government services digitally and enable digital literacy. The policy also aims to enhance business efficiency by simplifying the repatriation of export remittances, streamlining the functioning of electronic marketplaces, and reducing various administrative and transaction costs for SMEs. Additionally, the policy proposes including the e-commerce sector in the 'national integrated logistics plan' and enabling end-to-end logistics for SMEs by leveraging the Indian postal network to negotiate lower costs with international freight carriers. The National E-commerce policy further increases business efficiency by proposing industrial standards for the digital economy. Finally, this policy is also mindful of costs and export hurdles associated with e-commerce shipments of high-value items.

#### Policies Mitigating E-Commerce-Governance-Related Concerns

In the e-commerce context, the challenge to effectively evaluate the information gathered online could compromise transactional integrity (Oxley & Yeung, 2001). Indeed, e-commerce platforms provide opportunities for fraudulent reviews and other forms of opportunism. This is where institutions can play an essential role in reducing uncertainty by providing norms for acceptable behavior and the boundaries for legitimate actions (Peng et al., 2009), which can consequently promote firm’s international growth (Ahammad et al., 2021). The strength of the rule of law acts both as a powerful deterrent for opportunistic activities (e.g., online fraud) and a vehicle for generating greater transparency in the e-commerce context. Therefore, institutions can help provide greater governance in the e-commerce context and instill confidence (Oxley & Yeung, 2001). Building on these arguments, we outline how India's National E-commerce Policy initiative (Government of India, 2019a) reduces uncertainties and risks of opportunism for SMEs by providing guidelines regarding counterfeit products, consumer protection, compliant-handling, piracy, and security. The policy lays out specific guidelines regarding trademarks, copyrights, pirated products or content, and systems to redress complaints and disincentives to regulate the selling of counterfeits. The policy also advises against discrimination in publishing ratings and reviews and requires marketplaces to have systems to prevent fraudulent reviews from sellers and affiliates. Likewise, it provides for consumer protection and redressal of consumer grievances. Another example of how this policy reduces risk is by addressing data leaks, data theft, privacy, and transactional security. Furthermore, the policy requires local representation by all digital economy participants having access to data of Indians to ensure better governance by local authorities. All these measures help mitigate reputational, transactional, and unfair-competition risks to SME sellers.

#### Policies Mitigating Export Knowledge and Skill Barriers

There has been a rich stream of research investigating the role of export promotion policies and export assistance programs in reducing export barriers and enhancing the firm's internationalization and export performance (e.g., Leonidou et al., 2011). In this respect, the Indian Government's Digital SME scheme, Draft National E-commerce policy, Merchandise Exports from India Scheme (MEIS), and Service Exports from India Scheme (SEIS) (Government of India, 2019a, 2019b, 2019c) have been an instrument to enhance India's export competitiveness. Furthermore, these policies and schemes provide guidelines and incentives to facilitate SMEs' internationalization leveraging the e-commerce route. For example, The Digital SME scheme empowers and enables SMEs to effectively leverage ICT tools and applications and enhance their managerial and technical knowledge.

### Indian Government Policies Mitigating Industry-Level Barriers

Industry-level export barriers are associated with industry structure and the industry's competitive environment (Kahiya, 2013, 2018). More specifically, these barriers could include firms' size, restrictive industry regulations, price competition, limited technology, domestic and foreign competition, unfair trading practices, and industry concentration (Kahiya, 2013; Tesfom & Lutz, 2006). Many of these barriers are pertinent to the platform-based economy. Dominant global e-commerce platforms can wield significant market power over SME sellers due to their size, network effects, economies of scale, access to vast customer and market data, ability to control market prices, and unchallenged market position (OECD, 2019; UNCTAD, 2019). Therefore, platform-based models are at the center of anticompetitive concerns because platforms, unlike traditional companies, tend to accumulate power by network effects, leveraging third-party sellers, and switching costs. Platforms can also enhance their monopoly power by the value of monetary transactions and the data they control (UNCTAD, 2019). The vast amounts of data and information captured by e-commerce platforms could be manipulated using artificial intelligence, big data, and other cutting-edge technologies leading to market distortions. In this regard, India's National E-commerce Policy (Government of India, 2019a) analyzes the concentration of market power and associated market distortions from a 'data lens.' It proposes that the Indian Government has the right to seek disclosures of source code and algorithms.

The concentration of market power by e-commerce platforms also results from the first-mover advantage that gets amplified with the sheer scale and extensive network (Chen, 2019). Thus, National E-commerce Policy recognizes the significant disadvantage of second movers in this situation. Accordingly, the policy tries to minimize the threat of foreign e-commerce platforms to local SMEs by stipulating, “An e-commerce platform in which foreign investment has been made, therefore, cannot exercise ownership or control over the inventory sold on its platform. In this manner, foreign investment is not seen as a threat by small offline retailers of multi-branded products" (Government of India, 2019b, pp. 19–20). This policy mandate also addresses the conflict of interest concerns and resultant anticompetitive concerns arising when platform operators compete with other sellers on their platforms. In such cases, platform operators could act in an anticompetitive fashion by using pricing, sales, and other forms of data from sellers who also happen to be their downstream competitors (OECD, 2019).

Governments are also concerned about platforms using mergers and acquisitions to concentrate power. Accordingly, governments are reviewing their merger guidelines to consider the lower asset/turnover thresholds of digital start-ups (UNCTAD, 2019). The National E-commerce Policy (Government of India, 2019a) acknowledges this rising market dominance of e-commerce players and their use of mergers and acquisitions to consolidate power, reduce the competitive threat, and make it virtually impossible for second movers to enter the market. Hence, to protect and help small firms and start-ups grow and eventually compete in this environment, it gives such small firms the “infant industry status.”

Anticompetitive market behavior can also result in price-fixing and other distortionary practices. Another concern of the Indian Government is online marketplaces adopting business models and strategies that may be discriminatory. Recently, India's Competition Commission took note of allegations regarding certain e-commerce platform operators using exclusive selling arrangements and giving preferential treatment to some sellers (Singh, 2020). The National E-commerce policy cautions against platforms from favoring one or few sellers over others. Hence, the draft policy outlines that foreign direct investment (FDI) policy mandates fair and non-discriminatory treatment of all platform actors, including the SMEs. Furthermore, the policy advises that e-commerce advertising charges be regulated because platforms could leverage their monopoly power to extract high advertising charges that could disproportionately impact SMEs due to their shallow pockets. Thus, Indian Government policy guidance is addressing several anticompetitive threats in the local e-commerce environment.

The above discussion of Indian Government policies mitigating home-market and industry-level barriers and the emerging concerns make it a worthwhile pursuit to undertake a more in-depth qualitative investigation of these policies' impact on SME internationalization.

## Research Method

### Case Selection

Given the dearth of studies in this area, we employ an explorative lens to uncover insights regarding how Indian Government policies impact Indian SMEs’ platform-based internationalization efforts. More specifically, we use a case study method for this investigation. The case study method allows for inductive theorizing and accounting for complexities of context or local settings (Fletcher et al., 2018; Siggelkow, 2007).

Given this study's objectives, our primary criteria for case selection were — a) the SME should be engaged in e-commerce platform-based internationalization, and b) the SME should be knowledgeable about the e-commerce policy. Thus, purposive sampling was employed to include cases that inform our research questions regarding the impact of policy on platform-based internationalization of SMEs (Fletcher et al., 2018).

For implementing the case study method, an important question appertains to the number of investigated cases (Eisenhardt, 1989; Fletcher et al., 2018). Multiple case studies can provide detailed comparative insights, help confirm the information collected, and isolate case-specific idiosyncrasies. Scholars suggest that the number of cases should be determined by the nature of phenomena to be studied (Siggelkow, 2007) and, more importantly, the principle of theoretical saturation (Gioia et al., 2013). Based on these recommendations, we started with three case studies supplemented with two more case studies to achieve theoretical saturation. Accordingly, we included five different SME cases, and for further corroboration and development, we interviewed two government officials and one industry expert and referenced archival data. Table 1 provides a brief profile of the five cases selected for this study.

**Table 1:** Case Profile

	Case 1	Case 2	Case 3	Case 4	Case 5
Location	Jaipur	Delhi	Kolkata	Noida	Gurgaon
Establishment Year	2016	2017	2018	1996	2016
No of Employees	6	7	4	40	25
Total Turnover in 2019 in US$	267,000	90,000	81,920	2,112,676	7,042,253
Export Turnover in 2019 in US$	63,000	46,000	75,920	1,056,338	1,408,450
Key Export Markets	USA, UK, Canada, Middle East	Middle East, Australia, UK	USA, Canada, France, New Zealand, UK, Switzerland	USA, UK, Canada, Spain Singapore, Australia, Canada, France, Germany, Middle East	Middle East
Main E-commerce Channels	Amazon	Amazon & Own Website	Etsy	Amazon	Amazon & Own Website

### Data Collection and Validity

Primary data for each case study was collected through semi-structured interviews. In addition to case studies, we collected primary data from industry experts and government officials. Semi-structured interviews allowed us to explore the dynamics of policy and its implications for industry and its actors. We developed a priori interview guide, written in English to facilitate questioning our respondents. The interview guide also allowed us to yield cohesion, comparability, and continuity in narratives generated from each case (Harris, 2000). All interviews were conducted in the English language and recorded. However, recording permission for interviews with government officials was not granted, in which case notes were taken. Questions during the interview included exploring how Indian e-commerce policy initiatives are perceived regarding their ability to boost exports. For example, “how does the … schemes, namely, Merchandise Exports from India Scheme (MEIS) and Service Exports from India Scheme (SEIS) … help your firm (or your firm’s chances) to export globally?

To maintain ethical integrity and to protect the interest of participants, quotes are kept anonymous. Nonetheless, the authors had permission to go back to respondents for clarifications. A total of eight individuals were interviewed — five managers (one for each case study), two government officials, and one industry expert. Each respondent was interviewed twice, generating a total of sixteen interviews. On average, each interview lasted for approximately forty-five minutes. Finally, we mitigated possible bias by corroborating primary data with secondary sources. Collecting data from multiple sources helped triangulate data and enhance the transferability and trustworthiness of our findings (Munjal et al., 2021; Piekkari et al., 2009; Sinkovics et al., 2008).

### Data Analysis

We followed thematic analysis to identify commonalities and patterns in the data collected through interviews (Braun & Clarke, 2006). The analysis involved two authors comparing and interpreting the data by repeated analysis of interview recordings, transcriptions, reading and re-reading of notes, and confirming our understanding with the respondents. Thereafter, two authors open-coded the data independently to identify the codes and first-order categories. To enhance the study's trustworthiness, authors cross-checked the data and emerging themes; this process mitigated bias and objectivity issues. Simultaneously, we triangulated the data available from secondary sources, which enhanced our confidence in the interpretations and helped us overcome any bias originating from the interviewee’s side. This process thereby ensures poise and enhances our findings' transferability and trustworthiness (Piekkari et al., 2009; Sinkovics et al., 2008).

Besides, to ensure the validity of this process, we also followed an ‘independence and audit’ strategy for qualitative data analysis. Accordingly, an author who was not involved in the earlier coding process checked the codes independently. We also verified our data interpretations with the contact persons from case studies. Such corroboration of the findings helped us gain a complete understanding of the issue under scrutiny. This process of member check adds to the credibility of our findings (Shenton, 2004).

We identified patterns by comparing case studies to develop first-order concepts, second-order themes, and aggregate dimensions, as suggested by Gioia et al. (2013). Identifying the codes followed a “careful reading and re-reading” (Rice & Ezzy, 1999, p.258) of interview quotes and meticulously organizing them into codes. This process of analyzing data involved going back and forth between “academic pre-conceptualization and detailed empirical descriptions of emerging themes and topics” (Dawson, 1997, p. 390). Pettigrew (1997, p. 344) suggests the importance of these constantly iterating cycles as it involves “the real creative process of the research.” The process also involved two authors evaluating the correspondence among the first-order codes and jointly propounding the theoretical labels for classifying them under second-order themes. Finally, as we went through the coding iterations, we reached a stage at Case 5 wherein no new codes were identified. This led us to go over the entire dataset one more time to corroborate that no codes were missed. Thus, we believed we had delved deep enough to the point wherein the codes served as corroborating evidence rather than providing virgin insights. Eisenhardt (1989) and Gioia et al. (2013) have noted that such culmination of theme and conceptual development leads to theoretical saturation and signals that researchers could stop data collection.

The resultant initial open coding process allowed us to find commonalities among the respondent quotes across case studies and group them under first-order concepts or categories. The compendium of 16 first-order categories was then theoretically linked into labels representing seven second-order themes. Finally, the second-order themes were further distilled into three aggregate dimensions. In short, the first-order concepts identified commonalities within the data, and the second-order themes aggregated the commonalities with theoretical constructs yielding the overarching aggregate dimensions. Appendix-A captures our data structure showcasing the representative quotes, first-order categories, second-order themes, and aggregate dimensions.

## Findings and Discussion

The following discussion captures the insights based on our analysis and the resulting data structure, presented in Appendix-A. Findings are organized under the three aggregate dimensions: policies enhancing SME internationalization, burdensome compliance and logistics hurdles, and anticompetition challenges. Finally, this section also presents four propositions and a conceptual model for future testing and theorizing.

### Policies Enhancing SME Internationalization

Our findings suggest that India's Draft National E-commerce policy (Government of India, 2019a) has impacted operational efficiency and the modalities. For example, the policy has enhanced efficiency by simplifying administrative hurdles and improving interagency coordination. Expressly, the policy has enabled more integration among various agencies such as foreign trade, customs, foreign exchange, banking, and intellectual property, affecting e-commerce exports. These departments have different rules and regulations that firms engaged in e-commerce exports must comply with. For example, an official from the Director-General of Foreign Trade applauded the e-commerce policy for its distinguishing features. The officer said:

*India's e-commerce policy is world-class...But most importantly, our policy is designed to integrate different Government Departments: CBIC (Central Board of Indirect Taxes and Customs) for taxation, DGFT (Director General of Foreign Trade) for export/import policy, Department of Posts for product shipments. Banks are also integrated in the policy for issuing BRC (Bank Realization Certificate) to approve the realization of export turnover.*(Official, Director-General of Foreign Trade)

This view was echoed by other industry experts who took a macro view of the recent economic reforms and government policies and suggested a positive impact of these changes on exports undertaken by Indian SMEs. An official from the Federation of Indian Export Organizations (FIEO), an apex trade promotion organization of the Ministry of Commerce, and an expert on Indian e-commerce, with over 20 years of experience said:

*The Indian Government is doing right things to create a robust business environment…. India has moved up from 133*^*rd*^* to 63*^*rd*^* position in terms of ease of doing business. A lot of reforms like GST*^3^*and e-commerce policy are introduced to boost exports. Exports are exempt from GST. Policies are implemented to digitally refund any GST paid on exported items upon the filing of GST return to make MSME exports competitive.* (Official, FIEO)

The Indian Government has also made a concerted effort toward educating and training SMEs to adopt e-commerce for exports. Governments' educational efforts are being promulgated via the Draft National E-commerce policy (Government of India, 2019a), Digital SME scheme (Government of India, 2019b), and other initiatives by the Ministry of SME and Ministry of Skill Development and Entrepreneurship. Some quotes reflecting these educational efforts include:

*The Government of India push on digitization and digital footprint in business has pushed e-commerce a lot. E-commerce is a great livelier for MSMEs...* (Industry Expert)

*Government pays special attention on MSMEs through various schemes launched through the Ministry of Micro, Small and Medium Enterprises and the Ministry of Skill Development and Entrepreneurship*. (Official, FIEO)

Such policy measures primarily improve local firms’ awareness and confidence for internationalization (Suter et al., 2021). At the same time, it can significantly impact their operational efficiency, communication effectiveness, and, consequently, local firms' export efficiency (Leonidou, 2004). Appositely, our findings suggest that the Indian Government’s policies enhance global business opportunities for SMEs that are otherwise facing significant constraints regarding resources and knowledge necessary for internationalization. These insights chime with the exporting literature (Kahiya, 2013, 2018) regarding the Government's role in reducing internationalization barriers. Accordingly, we propose:

**Proposition-1**: E-commerce policies improving interagency coordination, procedural efficiency, and export education enhance export efficiency leading to platform-based SME internationalization.

As discussed in the earlier part of the paper, the Indian Government has proposed policy responses to manage anticompetitive threats in the Indian platform-economy context. The case insights further reinforce the importance of these policy actions. For example, respondents shared how India's National E-commerce policy has created a more level-playing ground for SMEs and mitigated some of their concerns regarding high advertising charges and favoring more significant sellers. Included below are some quotes reflecting these sentiments.

*I think the most important aspect of the e-commerce policy is that it provides a fair chance to small vendors like ours to sell our products. Earlier, e-commerce platforms were engaged in selling their own inventory through their own retail arms. They offered big discounts that we can't afford and paid high advertisement charges to promote their products on e-commerce platforms. Our products were neglected. It was really hard to create a place for ourselves in the e-commerce space. The policy restricts these things.... (Case -1)*

*The policy is regulating the industry somewhat well. There is a difference in terms of my experience…There was a discrimination, some kind of favoritism imposed on the listing by big sellers. If you search anything, their products were always on top of the list with big promotions. I think they also managed customer reviews to get their products up. I will say things are somewhat better now. (Case -2)*

*Small companies are facing difficulties in selling through online platforms as big companies blocked their ways by monopolization in various aspects until government policy came in force. (Case -3)*

We triangulated the case study data with secondary sources and expert interviews to better understand how the National E-commerce policy and recommendations regarding fairness and non-discrimination are perceived. For example, articles in *The Economic Times* outline how the draft policy addresses sellers' preferential treatment over others and other competition concerns. The quotes below highlight this issue:

*For the first time, small sellers on Amazon and Flipkart are offering prices similar to or lower than those set by large, preferred sellers. Two senior industry executives said the country's two largest online marketplaces have adopted this strategy by appointing multiple sellers for the top-selling products to comply with the revised foreign direct investment (FDI) for e-commerce.*

Source: The Economic Times (2019a)

*Online marketplaces Amazon and Flipkart have seen as much as a third of sales volume disappear on their platforms since the new foreign investment rules in ecommerce came into effect. The new regulations bar some of the business practices that foreign funded ecommerce companies followed, such as having a stake in companies that sold products on their platforms.*

Source: The Economic Times (2019b)

We further gained corroborative insights by speaking to the e-commerce expert who acknowledged that big retailers' market power was an issue and SMEs are still disadvantaged. However, the National E-commerce policy is still a work in progress that is still evolving. It is the first step by the Indian Government to regulate the evolving e-commerce industry in India.

*So earlier definitely it was a big issue tilting in favor of big players a lot... it has been brought down through regulations…but MSMEs continues to be at enough disadvantage.* Industry Expert)

Thus, based on these insights, it seems that the National E-commerce policy (Government of India, 2019a) has, to an extent, helped level the playing ground for various business actors. The policy has further reduced anticompetitive threats by tampering down the practices of preferring large vendors, charging high advertising charges, and using distortionary market practices. Accordingly, we propose:

**Proposition-2**: E-Commerce policies mitigating platform-based discriminatory practices and market dominance by large firms ease anticompetitive concerns leading to platform-based SME internationalization.

### Burdensome Compliance and Logistics Hurdles

The developments discussed in the previous section are positive steps that the Indian Government has undertaken to boost exports. However, SMEs face powerful bureaucracies when they try to exploit various e-commerce policy initiatives. There is support from the Indian Government, but the bureaucracy and procedural complexity are still challenging. For example, the following quote reflects this concern.

*There is support from Indian Government. But then to get that support to get the right information again is a big challenge in India. Again, you need to hire consultants for everything. There are rebates on certain HSN codes; there are no rebates on certain HSN codes. So, as a small business, I cannot afford to have 30 or so consultant… (Case-5)*

Similarly, the various Indian filling identification markers/numbers create a burdensome maze of technicalities and documentation for these small sellers as reflected in the following quote.

*These (identification markers) are all products of a time when things were not computerized,… And now when you have electronic everything… I don't think any person in this country needs to have more than an Aadhaar and GST number… (Case-4)*

Thus, this maze of rules, compliances, and complicated procedures dampen the operational efficiency of these new policy initiatives. For instance, compliances for exports are covered under the Customs, GST, and Banking laws. These can be categorized into pre-exporting and post-exporting compliances.

In the pre-export phase, SMEs are required to: a) obtain an Import Export Code (IEC); b) furnish a letter of undertaking (LUT) or Bond to export without payment of taxes to customs; c) have a robust Agreement/ Purchase Order for export of goods; d) file a shipping bill (bill of entry in case of air cargo) and details of export invoices; e) obtain an Authorized Dealer Code (ADC) for e-commerce exports.

In the post-exporting compliances are: a) file a shipping bill or bill of entry to the bank; b) obtain a foreign inward remittance certificate (FIRC) from importer's bank and filing this to their bank for obtaining a Bank Realization Certificate (BRC); and, c) file a BRC with the Director General of Foreign Trade (DGFT). These post-export compliances are necessary (for each export transaction) to declare that the export cycle is complete so that the tax credit and duty drawback can be claimed. There is a fixed time frame of one year within which these post-compliances are to be completed; otherwise, the transactions are not deemed as exports, and the exporter SME becomes liable to pay Goods and Services Tax (GST) on the transaction. The following quotes from our case respondents showcase the compliance burden imposed by this plethora of procedures.

*For many export transactions on e-commerce we get staggered remittances, e.g. Amazon pay us in staggered way for fulfillment by Amazon (FBA) sales. Even though this was one export order, we have to pay bank charges for BRC every time. Banks don't prioritize to spend time to issue BRC for each remittance either. This sometimes gets frustrating as my payments get delayed and cost keeps on increasing. Every Remittance in our bank account is being charged, which increases the cost of doing business. (Case -1)*

*On one occasion, I could not obtain the BRC within the stipulated time, as there was some delay in clearing the payment from the importers end. I ended up paying the GST and lost the GST credit without any fault. If I factor in GST in my price then my export becomes less competitive. (Case -2)*

*For each order, we have to prepare a lot of procedures and paperwork to file. These are tedious processes which many small entrepreneurs and artists are not able to fulfil. Subsequently, we have to file GST return to claim GST refunds, Foreign Inward Remmitence Certificate (FIRC), and BRC to realize bank payment and claim tax credits. Procedure of paperwork can be eased to some extent. This is certainly an area where Government should act. (Case -3)*

Another significant issue that hinders exports by Indian SMEs is the challenge of international product returns. Our analysis revealed that while the cost of reverse logistics curtails export margins, India’s National E-commerce policy (Government of India, 2019a) does not clarify provisions for the return of sales. Returns are presumed to be imported and therefore attract import duty.

Regarding managing returns …business model is not workable for most of the people unless they are sitting on a huge margin… it is not viable for MSMEs in India (Case -5)

If any goods are to be returned, the Government basically will charge you customs duty on those goods… (Case -4)

It was apparent that if SME sellers ship their returns back to India, they attract import duties. Furthermore, international product returns may face warehousing, shipping, and other reverse logistics charges. These reverse logistics charges and import duties hinder the export competitiveness of SME sellers. Some case respondents suggested that the Indian Government propel further and even subsidize Indian postal services to mitigate SMEs' logistics challenges. The following quote highlights this issue:

you know, Indian Postal Service also they should revamp…they need to subsidize it like what China has done… (Case -5)

Thus, the plethora of compliances, procedural complexities, and reverse logistics challenges create a complicated and burdensome route for SME exports. Accordingly, we propose.

**Proposition-3**: Burdensome compliance procedures and logistics hurdles attenuate the impact of e-commerce policies' export efficiency enhancements on platform-based SME internationalization.

### Anticompetitive Challenges

As discussed earlier, The National E-commerce policy (Government of India, 2019a) has reduced several anticompetitive threats, but many related challenges faced by SMEs are trammeling the policy effectiveness. The case insights reveal that some of these anticompetitive concerns regarding larger platform sellers are still not fully resolved. For example, the following quotes shed light on the changes in prominent retailers' modus operandi to maintain market power.

*The Online marketplaces are still dominated by large vendors. However, their business models have changed. They comply with the policy by not selling their own products… They are now acting like commission agents, selling goods of small vendors, like ours. I am being approached to sell through them and I am negotiating terms and conditions with them. I think they get subsidized rates for product listings, warehousing, and promotion. This is certainly an area where the Government should act. (Case -1)*

*Big retailers are still operating on marketplaces and I guess they are making most sales… As they are connected with the online marketplaces they dominate the show. You approach small sellers to sell through them. I was also being offered… but their terms and conditions are not in our favor. Case -2)*

Although these larger platform sellers provide SME sellers better platform visibility and branding, they may also be squeezing SME export margins. For example,

*…So because they are giving us that branding, and they know the systems of Amazon so they can sell better ... and ... they are saying that this is the margins you should be giving us on selling prices. And if I will be giving those margins to them then that is like eating up my own profit… (Case -3)*

When we probed this issue of big retailers with industry experts and referenced secondary data, this appears to not align with the policy. News reports published in leading newspapers suggest that the Confederation of All India Online Traders (CAIOT) seeks to probe online platforms’ and e-commerce firms’ business models for their unfair trade practices. Thus, stricter scrutiny of e-commerce players may be required to circumvent possible anticompetitive concerns. The industry expert echoed the view and said:

*there is a problem...platform is making policy for the platform and platform is also participating in business, so there is a disadvantage vis-à-vis other sellers…I am also a techie and I know what kind of artificial intelligence built into the system can give insight to platform if they have to take advantage of so there is a disproportionate advantage to the platform if they are also selling by having either their own inventory directly or through floated subsidiary or whatever…* (Industry Expert)

Counterfeit goods are another major issue that has an impact on the competitiveness of SME exporters. The Indian e-commerce policy addresses counterfeit goods and provides guidelines for confirming the genuineness of products. However, the case insights suggest the need to fix accountability and implement a robust system to end the listing of counterfeit goods on online platforms. Otherwise, counterfeit goods pose an unfair trade challenge to SMEs. The following quotes capture these insights.

It is not possible to control counterfeits. Happens everywhere in the world. It is difficult to identify counterfeits just by looking at pictures. Sellers also mixes counterfeit with originals making it difficult to identify…onus should be on seller. System of ranking and ratings may help. (Case -1)

Certainly, counterfeit goods are widely available online. You can't do much about them, so I don't bother too much. (Case -2)

*Our products have traditional Indian paintings, such as Madhubani, Kalamkari and many other forms of Indian Art done on textiles painted by artisans from different part of rural India. Each piece is uniquely done and cannot be replicated but sometimes people sell fake items in the name of Madhubani which naïve customers can’t differentiate… (Case-3)*

Our e-commerce expert seems to indicate that platforms have the responsibility to control counterfeit goods on their platform, and technology can be fruitfully employed to identify sellers who are indulged in such malpractices. The expert said eBay (2020) has a VeRO (Verified Rights Owner) program through which intellectual property owners could report listings or products that infringe on their intellectual property rights.

*Going forward more technology can be deployed to understand to give early alerts. Platforms saying, I do not own it, I cannot do anything is not the right behavior… their responsibility towards buyers... it is also true to bring discipline in sellers.* (Industry Expert)

A report in The Economic Times (2020a) suggests that the Indian Government sees a rise in the online sales of counterfeit goods as a 'worrisome' trend. Appropriately, the Government is looking at various ways to restrain counterfeit goods sold on e-marketplaces. The Confederation of All India Traders (CAIT) wants similar Indian e-commerce measures (The Economic Times, 2020b).

Thus, it seems plausible that while the Indian Government’s E-commerce Policies have, to an extent, addressed the platform economy's anticompetitive landscape, challenges regarding large platform sellers' market power and other unfair trade practices still loom and impede the full realization of India’s E-commerce Policy’s potential. Accordingly, we propose:

**Proposition-4**: Platform-specific unfair trading practices and the market dominance of large sellers attenuate the impact of anticompetition e-commerce policies on platform-based SME internationalization.

**Fig. 1:**
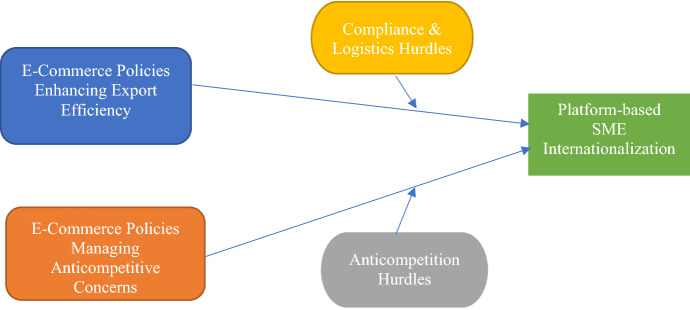
The Impact of India’s E-Commerce Policies on Platform-based SME Internationalization

In conclusion, our analysis and resulting propositions highlight how India’s National E-commerce Policy promoting export efficiency and mitigating anticompetitive concerns can enhance platform-based internationalization of SMEs. However, an unintended consequence of such policy reforms could be a more burdensome compliance landscape, which could thwart the intended outcomes of such policy reforms. Also, the policy cannot remain static in a dynamic platform-based economy context. Policymakers need to continually manage emerging anticompetitive threats to assist SME sellers in competing on a level playing field with large platform sellers. The conceptual model presented in Fig. 1 captures this aforesaid relationship between policy, SME internationalization, and anticompetitive and compliance hurdles.

## Conclusion

The desired outcome of any acceptable mercantile policy is to overcome barriers that constrain business activity. The literature shows the role of policy interventions in enhancing firm productivity and export intensity (Bose et al., 2020). Indeed, our discussion on various Indian Government policy initiatives highlights the extent to which the Government is engaged in mitigating home market and industry-level barriers to SMEs’ platform-based internationalization. However, our study also emphasizes that there are various policy challenges that governments need to address to enhance the e-commerce-based internationalization of SMEs. Accordingly, to elaborate on these challenges and their implications, we outline the policy and theoretical implications and limitations and future research.

### Policy Implications

One of the key policy implications from this study is that governments need to specifically focus on reducing the compliance and bureaucratic hurdles faced by smaller firms. The Indian Government policies have yet to address the issue of excessive paperwork and compliance hurdles. The case insights highlight the compliance barriers and the excessive paperwork SMEs must undertake to avail specific export incentives and comply with the laws. This situation is further exacerbated from a broader economic perspective as Indian businesses have to deal with around 1,500 laws, 69,000 compliances, and 6,600 filings every year (Zee Business, 2020). Indeed, these compliances are more difficult for the SME sector in comparison to larger firms. Notwithstanding, the e-commerce platforms exploit compliance-related gaps in the policy by providing intermediary or third-party services to help SMEs with export-related documentation.

While excessive compliance may have remained, the policy has undoubtedly provided a window of opportunity to the Indian SMEs to internationalize via e-commerce platforms. As indicated in our findings, e-commerce platforms provide foreign market entry without much capital investment, which can be a valuable takeaway for SME managers to test their products’ suitability in foreign markets.

Nevertheless, e-commerce platforms are still tilted in favor of larger sellers rather than SME sellers. Large platform-sellers are better positioned to afford platform-based advertising and marketing opportunities, leading to visibility and customer acquisition. Sometimes to gain customers and visibility, smaller sellers sell via these larger sellers, which reduces their profit margins. Smaller sellers are further disadvantaged when it comes to international product returns. Reverse logistics charges and import duties can make it difficult for SME sellers to reclaim their customer returns, reducing their profit margin. Several quotes in our data structure (Appendix-A) reflect these concerns. Thus, to enhance platform-based SME internationalization, the Indian Government's Policy needs to evolve to mitigate emerging and future anticompetitive concerns.

### Theoretical Implications

Besides the scrutiny of the policy and recommendations, our study highlights two key theoretical implications. First and foremost, it illustrates the co-evolution of policy and business strategy and identifies the industry and home-country barriers as triggers for such evolution. Scholars (Cantwell et al., 2010; McGaughey et al., 2016) argue that the relationship between businesses and institutions is intertwined, as institutions co-evolve with the evolution of the firm. Our study adds to this stream of knowledge by revealing that changes in the technological domain and consequent business models (e.g., online marketplace model) trigger the process of such co-evolution.

Secondly, the study highlights the role of local context in firms' internationalization, which tends to be taken for granted in IB research (Meyer et al., 2011). Local context shapes the structure and strategy of the firm, which affects its internationalization (Buckley & Munjal, 2017). Our study specifically highlighted the role of Indian Government policies in directly (e.g., by increasing the value of export undertaken through e-commerce and automatic GST refunds on exports) and indirectly (via regulating the conduct of e-commerce platforms) promoting SME internationalization.

### Future Research and Limitations

Indian online SME sellers still face various cross-country tariff and non-tariff barriers. The Indian Government has initiated free trade agreements to enhance SMEs’ export opportunities, but how these trade agreements enhance platform-based SME sellers' price and export competitiveness could be a fruitful area for future investigation.

Another exciting avenue for future research is to synthesize strategic choice theory and institutional theory to understand better how platform actors comply, navigate, and influence policy. The strategic choice theory may be a beneficial lens to explore how organizations or dominant coalitions respond to institutional constraints in the context of the platform-based economy (Child, 1997; Oliver, 1991). Based on our analysis, there is emerging evidence of platform actors engaging with the Indian Government to shape policy guidelines addressing issues such as safeguarding against counterfeit products and fake reviews, minimizing export hurdles, and improving SMEs' export education. For example, Gopal Pillai, VP of seller services at Amazon India, shares that Amazon's close cooperation with the Indian Government resulted in post offices accepting export boxes, reduction of export forms from fourteen to three, and increase in export subsidy from two percent to four percent (Rakshit, 2019). Thus, to further enrich our understanding of the role of policy on platform-based SME internationalization, we need to study how firms manage and comply with institutional constraints and how they actively shape the institutional environment (See Fig. 1)

Finally, as Gioia et al. (2013) suggested, propositions in qualitative research can serve as a roadmap for future theory building. We hope our propositions will engender further investigations of the impact of e-commerce policies on platform-based SME internationalization. Last but not least, our study has a fair share of limitations as the data is drawn from a limited number of case studies. Future research can test our ideas by collecting survey-based data from a large number of SMEs and possibly triangulate primary and secondary data for confirming or extending our findings. Scholars can also check the efficacy of e-commerce policy by comparing e-commerce policies from different countries and make recommendations for further evolution.

## Supplementary Information

Below is the link to the electronic supplementary material.

Supplementary file1 (DOCX 35 KB)

## Footnotes

1 E-commerce platforms in the context of this study are "*online marketplace operators bringing together many buyers and sellers by leveraging information, technology, governance mechanisms, and complementors to enable exchange and value creation in a global arena.”.*

2 According to Brynjolfsson et al. (2008) ‘Scale without mass’ is the rapid scaling of processes and innovations due to dwindling per unit cost for processing, storing, replicating and transmitting data (OECD, 2019).

3 The term GST in the quote refers to ‘Goods and Services Tax’ which is equivalent to the Value Added Tax ‘VAT’ in the United States and Europe.
