# Supplementary material for: Platform-Based Internationalization of Smaller Firms: The Role of Government Policy
Source: Manag Int Rev. 2022 Nov 26;63(1):91–115. doi: 10.1007/s11575-022-00492-z (PMC9702645; doi:10.1007/s11575-022-00492-z)
Supplement: Supplementary file 1 — Supplementary file1 (DOCX 35 KB) [file 11575_2022_492_MOESM1_ESM.docx]

**Platform-Based Internationalization of Smaller Firms: The Role of Government Policy**

**Appendix-A: Data Structure: First Order Categories, Second Order Themes, and Aggregate Dimensions**

| **Some Representative Quotes (abridged)** | **First-order Categories** | **Second-order Themes** | **Aggregate Dimensions** |
| --- | --- | --- | --- |
| A1: *Policy is designed to integrate different Government Departments.*  *A2: Banks are integrated in the policy for issuing BRC.*  *B1: Policies are implemented to digitally refund any GST paid on exported items.*  *B2: A lot of reforms, such as GST and e-commerce policy, are introduced to boost exports.*  *B3: GST, for example, is bringing lot of transparency in business.*  *C1: Government pays special attention on MSMEs through various schemes launched through the Ministry of Micro, Small and Medium Enterprises and the Ministry of Skill Development and Entrepreneurship.*  *C2: Digital India policies are helping MSMEs.*  *C3 Government of India push on digitization and digital footprint in business has pushed e-commerce a lot.* | A: Policies enhancing interagency cooperation  B: Policies streamlining export procedures  C: Policies promoting export training | Policies for export efficiency | Policies enhancing SME Internation-  alization |
| D1: *Earlier, e-commerce platforms were engaged in selling their own inventory… The policy restricts these things.*  *D2: There was a discrimination… on the listing by big sellers…I will say things are somewhat better now.*  *D3: Now there are certain set of regulations on these big companies. So, they now have to keep their feet really cautiously.*  *E1: Small companies are facing difficulties …as big companies blocked their ways by monopolization…until government policy came in force.*  *E2: It provides fair chance to small vendors.*  *E3: Now things are better, but the big retailers are still there* | D: Policies mitigating discriminatory practices by platforms  E: Policies restricting market dominance | Policies managing anticompetitive practices |  |
| *F1: Every Remittance in our bank account is being charged.*  *F2: Staggered remittances on a single export transaction attracts multiple bank charges.*  *F3: Banks don't prioritize BRC for each remittance.*  *G1: We have to file GST return to claim GST refunds and FRIC, and BRC to realize bank payment and claim tax credits.*  *G2: I could not obtain the BRC within stipulated time… I ended up paying the GST and lost the GST credit without any fault.*  *G3: Our profits are getting blocked into the credit GST ledger.* | F: Issues with bank compliances  G: Issues with tax-related compliances | Plethora of Compliances | Compliance & logistics hurdles |
| *H1: It takes a lot of time you know, to push them really hard …. there’s no clear-cut policy.*  *H2:People in Government…still working as government employee... they don't answer properly, …pick up the phones… no proper online services.*  *H3: See it takes away lot of energy and time in meeting government compliances, every time you export.*  *H4: The bureaucracy is time consuming and expensive.*  *I1: For each order we have to prepare a lot of procedures and paperwork.*  *I2: there is support from Indian Government. But then to get that support is a big challenge.*  *I3: You need to hire consultants for everything* | H: Psychic costs of compliance  I: Procedural complexity | Bureaucratic challenges or Red tape |  |
| *J1: If any goods are to be returned, Government charges customs duty.*  *J2: We have to pay the duty which is quite high…, for garments there is a duty say 20-25% for imports.*  *K1: The return shipment is a major challenge… There has to be some mechanism of getting the stocks back to India.*  K2: Regarding returns*…business model is not workable…unless they are sitting on a huge margin... it is not viable for MSMEs in India.*  *K3: Indian Postal Service... should revamp….they need to subsidize it like what China has done.* | J: Customs duties on returns  K: Reverse logistics challenges | Int. product return Issues |  |
| *L1: Amazon competition is severe….the consumer surplus Amazon can mop up right by doing the auction.*  *L2: Our products were neglected. It was really hard to create a place for ourselves in the e-commerce space.*  *L3: Amazon is charging high commissions I agree, but that is purely because it's a monopolistic market.*  *M1: If you search anything, their products were always on top of the list with big promotions.*  *M2: They offered big discounts that we can't afford.*  *M3: We paid high advertisement charges.*  *N1: Sellers mix counterfeit with originals making it difficult to identify*  *N2: Counterfeit goods are widely available online. You can't do much about them.*  *N3: There are so many fake goods available on the online market places.* | L: Inventory and product listing concerns  M: Discounts and advertising concerns  N: Counterfeit concerns | Unfair trading practices | Anticompe-tition hurdles |
| *O1: If Government comes up with some more regulations, then it is beneficial for small sellers…the big sellers are getting bigger, small sellers are getting out of the market slowly.*  *O2: It's not at all a level playing field, big players get more chances.*  *O3: Big retailers’ main target is to wipe out these small businesses from the market and they are doing it the wrong way... this has to be stopped.*  *P1: There was a discrimination, some kind of favoritism imposed on the listing by big sellers.*  *P2: Because they are giving us that branding, and they know the systems of Amazon…they can sell better… and they are saying that this is the margins you should be giving us.*  *P3: Their terms and conditions are not in our favor.* | O: Big retailer’s power  P:Discrimination issues related to big retailers | Market power of big retailers on the platform |  |

Note: Following Gioia methodology (Gioia et al., 2013): The Appendix shows the data structure based on 47 quotes from our coding, 16 first-order categories, 7 second-order themes, and 3 Aggregate dimensions
